# Acoustic characteristics of fricatives, amplitude of formants and clarity of speech produced without and with a medical mask

**DOI:** 10.1111/1460-6984.12705

**Published:** 2022-02-15

**Authors:** Duy Duong Nguyen, Antonia Chacon, Christopher Payten, Rebecca Black, Meet Sheth, Patricia McCabe, Daniel Novakovic, Catherine Madill

**Affiliations:** ^1^ Voice Research Laboratory Faculty of Medicine and Health Susan Wakil Health Building Camperdown Campus The University of Sydney Sydney NSW Australia; ^2^ National Hospital of Otorhinolaryngology Hanoi Vietnam; ^3^ The Canterbury Hospital Campsie NSW Australia; ^4^ Sydney Voice and Swallowing St Leonards NSW Australia

**Keywords:** acoustic analysis, formant amplitude, fricative signal, speech clarity, vowel amplitude

## Abstract

**Background:**

Previous research has found that high‐frequency energy of speech signals decreased while wearing face masks. However, no study has examined the specific spectral characteristics of fricative consonants and vowels and the perception of clarity of speech in mask wearing.

**Aims:**

To investigate acoustic–phonetic characteristics of fricative consonants and vowels and auditory perceptual rating of clarity of speech produced with and without wearing a face mask.

**Methods & Procedures:**

A total of 16 healthcare workers read the Rainbow Passage using modal phonation in three conditions: without a face mask, with a standard surgical mask and with a KN95 mask (China GB2626‐2006, a medical respirator with higher barrier level than the standard surgical mask). Speech samples were acoustically analysed for root mean square (RMS) amplitude (*A*_RMS_) and spectral moments of four fricatives /f/, /s/, /ʃ/ and /z/; and amplitude of the first three formants (A1, A2 and A3) measured from the reading passage and extracted vowels. Auditory perception of speech clarity was performed. Data were compared across mask and non‐mask conditions using linear mixed models.

**Outcomes & Results:**

The *A*_RMS_ of all included fricatives was significantly lower in surgical mask and KN95 mask compared with non‐mask condition. Centre of gravity of /f/ decreased in both surgical and KN95 mask while other spectral moments did not show systematic significant linear trends across mask conditions. None of the formant amplitude measures was statistically different across conditions. Speech clarity was significantly poorer in both surgical and KN95 mask conditions.

**Conclusions & Implications:**

Speech produced while wearing either a surgical mask or KN95 mask was associated with decreased fricative amplitude and poorer speech clarity.

**WHAT THIS PAPER ADDS:**

**What is already known on the subject**

- Previous studies have shown that the overall spectral levels in high frequency ranges and intelligibility are decreased for speech produced with a face mask. It is unclear how different types of the speech signals that is, fricatives and vowels are presented in speech produced with wearing either a medical surgical or KN95 mask. It is also unclear whether ratings of speech clarity are similar for speech produced with these face masks.

**What this paper adds to existing knowledge**

- Speech data collected using a real‐world, clinical and non‐laboratory‐controlled settings showed differences in the amplitude of fricatives and speech clarity ratings between non‐mask and mask‐wearing conditions. Formant amplitude did not show significant differences in mask‐wearing conditions compared with non‐mask.

**What are the potential or actual clinical implications of this work?**

- Wearing a surgical mask or a KN95 mask had different effects on consonants and vowels. It appeared from the findings in this study that these masks only affected fricative consonants and did not affect vowel production. The poorer speech clarity in these mask‐wearing conditions has important implications for speech perception in communication between clinical staff and between medical officers and patients in clinics, and between people in everyday situations. The impact of these masks on speech perception may be more pronounced in people with hearing impairment and communication disorders. In voice evaluation and/or therapy sessions, the effects of wearing a medical mask can occur bidirectionally for both the clinician and the patient. The patient may find it more challenging to understand the speech conveyed by the clinician while the clinician may not perceptually assess patient's speech and voice accurately. Given the significant correlation between clarity ratings and fricative amplitude, improving fricative signals would be useful to improve speech clarity while wearing these medical face masks.

## INTRODUCTION

Face masks are used for preventing droplet and/or aerosol‐based transmission of infection (Long et al., 2020). Two main types of medical masks offer different levels of protection. Fluid‐resistant medical masks (surgical masks) are worn both by healthcare workers (HCW) and the community. They fit loosely on the face and are designed to reduce the spread of large droplets. Medical respirators including the N95 type are high‐performance filtering masks designed to be tightly attached and prevent against inhalation of small airborne particles (Cook, 2020). The World Health Organization (WHO) recommends the use of surgical masks in lower risk situations and respirators in high‐risk contexts, such as frontline HCWs who may be directly involved in aerosol‐generating procedures (Cook, 2020). However, wearing a mask may negatively affect the physiological and psychological performance of the wearer (Johnson, 2016).

Previous studies have shown an attenuation in high‐frequency spectral energies in speech produced with a face mask. Using a model experiment, Goldin et al. (2020) found that the surgical mask and the N95 mask attenuated the sound levels at frequency regions between 2 and 7 kHz. Corey et al. (2020) demonstrated that all types of face masks attenuated frequencies > 1 kHz. Magee et al. (2020) showed that different mask types affected different spectral regions with the N95 mask impacting > 3 kHz and surgical and cloth masks > 5 kHz. Nguyen et al. (2021) showed that spectral levels of connected speech were significantly lower in the 1–8 kHz frequency range in surgical and KN95 masks. Atcherson et al. (2017) showed that the total root mean square (RMS) of speech was significantly lower in mask‐wearing than non‐mask conditions. However, no study has investigated the specific acoustic characteristics of speech units such as fricatives and vowels in mask wearing.

The high‐frequency spectral energy contains information needed for the recognition of vowels (Donai & Paschall, 2015) and voiceless and voiced fricative consonants (Vitela et al., 2015). Formant amplitude plays an important role in vowel perception (Kiefte et al., 2010) and may relate to the naturalness and phonetic quality of vowels (Jacewicz, 2005). Spectral amplitude is important in identifying voiceless fricative consonants (Behrens & Blumstein, 1988a), the frequency of which are within the high frequency regions (e.g., the centre of gravity value is 6133 Hz for English fricative /s/; Jongman et al., 2000). To date, there is a limited understanding of the acoustic characteristics of fricative amplitude and higher formant amplitude in speech produced in mask wearing. Llamas et al. (2009) showed misperceptions of speech in mask‐wearing resulting from confusion of stop consonants (e.g., /t/∼/θ/), misperception of place of articulation of stop consonants and place of articulation of fricatives, for example, /f/∼/θ/. They found that speech produced with a surgical mask had lower amplitude of vowel formants and fricative energy > 3.2 kHz. However, they only provided visual illustration of their observations and did not provide quantitative data on speech spectral characteristics. It seems necessary to investigate how spectral amplitude measures relevant to fricative consonants and vowels are presented while wearing a face mask. Fricatives can be examined using spectral amplitude for example, RMS amplitude and spectral moment measures (centre of gravity, standard deviation, skewness and kurtosis) (Jongman et al., 2000). Spectral levels of vowels can be quantified using formant amplitude (Kiefte et al., 2010).

The degraded signals in speech produced while wearing a face mask can affect speech perception (Bandaru et al., 2020). Radonovich et al. (2010) showed that the N95 mask resulted in a mean (SD) of Modified Rhyme Test score of 83 (16.2)% compared with 92 (5.8)% in non‐mask controls. Bandaru et al. (2020) found an increased speech reception threshold and decreased speech discrimination score whilst the N95 mask was worn. Mendel et al. (2008), however, showed that the use of a standard surgical mask did not have unfavourable effects upon speech understanding in both the normal‐hearing and hearing‐impaired listeners. No study has examined the perception of clarity of speech produced with wearing a face mask.

Speech clarity can refer to ‘clear speech’, which is a speaking style adopted in difficult communication situations (Uchanski, 2005) and is out of the scope of this study. On the other hand, speech clarity also indicates the overall quality of the speech sounds in conversational speaking style, and this was the focus of this study. In this perspective, speech clarity, or the perception of how clear a speaker's speech is to the listener, is a commonly used term, but is non‐specific and rarely defined in research or clinical literature (Liu et al., 2003; Reinhart & Souza, 2016). Some authors have offered suggestions of speech precision (Knollman‐Porter & Burshnic, 2020) as an equivalent descriptor of the phenomenon which is differentiated from acoustic speech clarity and intelligibility measures such as the Speech Transmission Index (STI), a physical metric that is well correlated with the intelligibility of speech degraded by additive noise and reverberation (Goldsworthy & Greenberg, 2004), and the measured 50 ms early‐to‐late sound energy ratio, C50, an index used to quantify speech intelligibility in enclosed spaces (Marshall, 1995). In this paper, we assume that clarity is one of many dimensions of speech quality such as ‘naturalness’, ‘clarity’, ‘brightness’ or ‘pleasantness’ (Loizou, 2011) that can be quantifiable using a relevant perceptual rating scale. The perception of speech clarity has been examined using different rating schemes. Some authors have used rating scales to quantify speech clarity. For example, Tasko and Greilick (2010) used a computer‐based slider scale where raters compared clarity of word pairs and made judgment by moving the slider from the mid‐point of the scale toward the clearer stimulus. Reinhart and Souza (2016) used a seven‐point Likert scale, with 1 representing ‘completely unclear’ and 7 ‘completely clear’.

Given that medical masks introduce certain barrier levels in terms of filtering, inhalation and exhalation resistance, we hypothesized that (1) speech produced whilst wearing a medical mask would have different characteristics of fricative and vowel amplitude of higher formants (i.e., the second and third formants) compared with non‐mask condition; and (2) levels of speech clarity would be different between mask and non‐mask conditions. The aims of the present study were (1) to evaluate amplitude and spectral moment measures of fricative consonants in speech produced with and without wearing a medical face mask; (2) to quantify vowel formant amplitude in speech with and without a medical face mask; and (3) to examine the perception of the clarity of speech produced without and with wearing a medical face mask.

## METHODS

### Ethical approval

This study was approved by the Human Research Ethics Committee of the University of Sydney (project number 2020/399). Informed consent was obtained from all participants to participate in this study. All methods used in the present study were performed in accordance with the relevant ethical guidelines and regulations.

### Speech sample recording

#### Speakers

A total of 16 speaker HCWs (12 females, four males) took part in this study with mean age of 43 years (range = 24–61), including two otolaryngologists, 13 practising speech–language pathologists (SLP), and one registered nurse working in an ear, nose and throat (ENT) clinic. Inclusion criteria were English speakers, non‐smokers, with no self‐reported voice or hearing problems at the time of the study.

#### Medical mask types in this study

The present study focused on surgical masks and KN95 (China GB2626‐2006), which is a medical respirator. The surgical mask used in the present study had characteristics as follows: brand name: Strapit mask (Strapit HQ, 2021), level 2 barrier and fluid resistant. Characteristics of level 2 barrier masks are as follows (Australian Standard, 2015): bacterial filtration efficiency ≥ 98%, differential pressure < 5.0 mmH_2_O/cm^2^, resistance to penetration by synthetic blood, and minimum pressure = 120 mmHg. The KN95 mask (brand name RNA Pharmaceuticals KN95 face mask) had filtering and fitting characteristics as follows: filter performance ≥ 95%; flow rate = 85 L/min; inhalation resistance ≤ 350 Pa; exhalation resistance ≤ 250 Pa; and total inward leakage < 8% (3M, 2020). The total inward leakage indicates the amount of an aerosol that enters the mask via both filter penetration and face‐seal leakage (3M, 2020).

#### Voice recordings

This study used real‐world rather than laboratory‐controlled settings to reproduce the speech sound typically heard in clinical settings whilst a HCW is wearing a medical mask. Speech sample recordings were performed in a quiet room or soundproof booth at the participants’ respective clinics as social distancing measures during the COVID‐19 pandemic prohibited the use of the same room. Participants were required to read the Rainbow Passage (Fairbanks, 1960) in three conditions in the same recording session: (1) not wearing a mask; (2) wearing a surgical mask; or (3) wearing a KN95 mask. The order of conditions was randomized across speakers. Speakers were required to use their habitual, modal phonation at their comfortable pitch and intensity and did not use the ‘clear speech’ style to minimize intra‐speaker variability in phonation and potential compensation while wearing a mask. When wearing these masks, participants were required to use the highest level of fitting to ensure maximal barrier level. They were required to press the nose metal bar so that it fit tightly to the nose contour. The straps of the mask were securely placed behind the auricles and the lower side of the mask was pulled fully downward so that it covered the chin completely. It has been known that in unfavourable/challenging speaking conditions, speakers may adapt a phonation style that helps improve clear phonation (Krause & Braida, 2004). Therefore, we required participants to maintain similar habitual voice in terms of pitch, loudness, and speaking style throughout recording sessions both with and without a mask to minimize intra‐speaker variability in voice production.

All voice signals were captured using an AKG C520 cardioid ear‐mounted microphone placed at a constant distance of 6 cm, 45^o^ off the mouth axis with analogue‐to‐digital conversion via a professional external sound card (Roland Quadcapture) at 44.1 kHz and 16‐bit resolution. The signals were processed and saved to a laptop computer using the Audacity sound editing software in *.wav format.

Given that voice recordings took place in different clinic rooms with different levels of background noise, audio files were examined for signal‐to‐noise ratio (SNR) using a Praat script called Speech‐to‐noise ratio/Voice‐to‐noise ratio v.01.01 (Maryn, 2020). Only samples with a SNR > 30 dB were used for auditory–perceptual and acoustic analyses (Deliyski et al., 2005).

### Acoustic analyses

#### RMS amplitude (*A*_RMS_) of fricatives

The *A*_RMS_ was used because this has been frequently examined to characterize English fricatives (Jongman et al., 2000). The amplitude of the fricative signal has also been considered an important cue to perceive the place of articulation in fricatives and hence the accuracy of fricative consonant production (Behrens & Blumstein, 1988a). This measure was obtained from the following fricatives: /f/ (labiodental), /s/ (alveolar), /ʃ/ (palato‐alveolar) and /z/ (alveolar) because these represent different places of articulation (Jongman et al., 2000). The /s/ and /ʃ/ are characterized by well‐defined spectral shapes compared with labio‐dental /f/ and dental /θ/ which have a relatively flat spectrum without a clear dominant peak (Jongman et al., 2000). Dental fricatives (e.g., /θ/) were not used as they have shorter noise duration and similar spectral characteristics as /f/ in terms of spectral peak location, spectral mean, and noise amplitude (Jongman et al., 2000). Fricatives /s/ and /ʃ/ were used for specific reasons. These fricatives have higher amplitude and longer duration than other voiceless fricatives, for example, /θ/ and /f/ (Behrens & Blumstein, 1988b) which would make it easier to reliably identify and extract them from connected speech.

**FIGURE 1:**
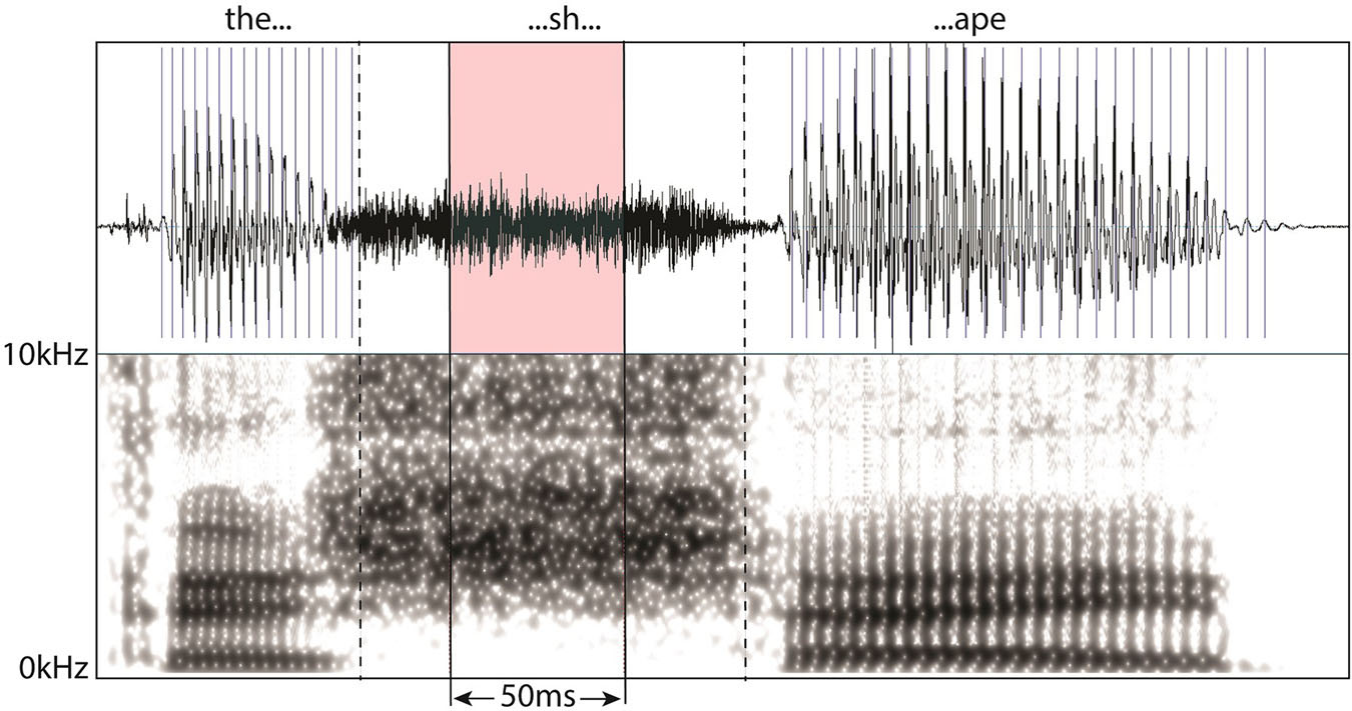
Wide‐band spectrogram illustrating the extraction of the fricative /ʃ/ in Praat. Vertical dashed lines indicate boundaries of this consonant [Colour figure can be viewed at wileyonlinelibrary.com]

First, the signals were high‐pass filtered at 1 kHz in Audacity with 6‐dB roll‐off per octave to remove any potential trace of voicing due to the pre‐ and post‐vocalic environment of the fricative (Martel‐Sauvageau et al., 2021) and to minimize low‐frequency energy which could interfere with detection of zero‐crossings due to the turbulent source (Maniwa et al., 2009). The fricative /f/ was edited from the word ‘form’ in ‘they act as a prism and form a rainbow …’; /ʃ/ was extracted from ‘*shape*’ in ‘These take the shape of a long round arch …’; /z/ was edited from ‘horizon’ in ‘its two ends apparently beyond the horizon …’; and /s/ was extracted from ‘say’ in ‘his friends say he is looking for the pot of gold …’. The boundaries of the consonants were identified visually using acoustic waveform and spectrograms in Praat 6.1.40 (Boersma & Weenink, 2018) (Figure 1) and by listening to the sample. Fricative signals were defined as having the following criteria: (1) characteristic waveform with zero‐crossing; and (2) high‐frequency noise energy in the narrow‐band spectrogram. The middle 50 ms segment was extracted from the centre of these fricatives for acoustic analysis. Onset and offset segments were excluded as for these fricatives, the onset (immediately before voicing onset) and offset have lower amplitude than the middle (Behrens & Blumstein, 1988b), hence extracting the middle segment would increase the probability of capturing the amplitude peaks of the fricative noise signals. The edited fricatives underwent a fast Fourier transform and was analysed in Praat in the frequency range 1–10 kHz.

The *A*_RMS_ over the time interval *t*1 ≤ *t* ≤ *t*2 was defined using the formula (Boersma & Weenink, 2018):

(1) $$A_{RMS}=\sqrt{\frac{1}{\left(t2-t1\right)}\int_{t1}^{t2}{\left[x\left(t\right)\right]}^{2}dt}$$

where *x*(*t*) is the amplitude of the sound. The squared amplitude is averaged over the time *t*, then the square root is calculated for the averaged squared amplitude. *A*_RMS_ was converted from Pascal (Pa) unit in Praat to sound pressure level (SPL) in dB using the formula:

(2) $$dB\;SPL=20log_{10}\left(P/P_{0}\right)$$

where *P* was the *A*_RMS_ value; and *P*_0_ = 20 μPa, which was the reference value.

#### Spectral moments of fricatives

Centre of gravity (Hz), standard deviation (SD; Hz), skewness, and kurtosis were obtained from Praat because these measures have been used to characterize English fricatives (Jongman et al., 2000). Previous research has shown that spectral moments of fricatives are important correlates of clear spoken speech (Maniwa et al., 2009). Measuring these would not only investigate the fricative characteristics in non‐mask and mask conditions but also serve as a post‐hoc test to clarify whether this speaking style occurred in the presence of a face mask.

#### Amplitude of the first three formants

Amplitude of vowels in context can be quantified using formant amplitude (Jacewicz & Fox, 2008). Therefore, this study measured amplitude of the first three formants (hence A1, A2 and A3) from two types of tasks: The Rainbow Passage, and vowels edited from this passage: /ɐː/ in ‘arch’, /ɪ/ in ‘many’ and /ʊ/ in ‘two’. The vowels were extracted using Praat by listening and identifying waveform and spectrogram characteristics associated with the required vowel. They represent primary cardinal vowels with the highest and most forward tongue position (/ɪ/), highest and most backward tongue position /ʊ/ and lowest tongue position (/ɐː/) (Ladefoged, 2011). All measurements were implemented automatically at every 1 ms for voiced segments with a window length of 25 ms (Jacewicz, 2005) using a MATLAB‐based program called VoiceSauce (Shue et al., 2009). The amplitudes were automatically corrected for the co‐articulatory effects of the surrounding formants and linguistic units (Shue et al., 2009). The highest formant amplitude within this window length was obtained. Settings were as follows: min fundamental frequency (F0) = 75 Hz, and max F0 = 400 Hz; pre‐emphasis = 0.96; and linear predictive coding (LPC) order = 12. Data points with zero values were deleted. To minimize intra‐ and inter‐speaker variability in phonation related to connected speech production, all amplitude measures were normalized so that the minimal and maximal values were 0 and 1, respectively.

#### Reliability of acoustic analyses

A co‐author repeated the file editing and measurement process on both /s/ and /ʃ/. Table 1 shows results of intraclass correlation coefficients calculated for the two fricative consonants in the non‐mask condition.

**TABLE 1:** Intraclass correlation coefficient (ICC) for interrater reliability of spectral measures for two fricatives /s/ and /ʃ/ in non‐mask

		**/s/**	**/ʃ/**
**Spectral measures**	**Measures**	**ICC (95% CI)**	**ICC (95% CI)**
Root mean square (RMS)	SM	0.997 (0.991–0.999)	0.999 (0.997–1.000)
	AM	0.998 (0.996–0.999)	1.000 (0.999–1.000)
Centre of gravity	SM	0.998 (0.994–0.999)	0.996 (0.988–0.998)
	AM	0.999 (0.997–1.000)	0.998 (0.994–0.999)
Standard deviation	SM	0.987 (0.962–0.995)	0.966 (0.905–0.988)
	AM	0.993 (0.981–0.998)	0.983 (0.950–0.994)
Skewness	SM	0.968 (0.910–0.989)	0.991 (0.974–0.997)
	AM	0.984 (0.953–0.994)	0.995 (0.987–0.998)
Kurtosis	SM	0.991 (0.976–0.997)	0.998 (0.995–0.999)
	AM	0.996 (0.988–0.999)	0.999 (0.998–1.000)

*Notes*: AM, average measures; CI, confidence interval; and SM, single measures.

All *p*‐values < 0.001.

### Auditory–perceptual analyses

#### Raters

Seven raters who demonstrated good intra‐rater reliability (see below) were included in the study. Listeners were invited via email advertisement sent to an international professional network of SLP and ENT specialists. Inclusion criteria included: (1) working with voice patients as speech–language pathologists, voice specialists, or laryngologists; and (2) normal hearing at the time of the study. Only specialists were involved to provide expert's ratings of speech clarity which would be difficult for the naïve listeners to evaluate.

#### Perceptual rating scale

In this study we were interested in the ratings of clarity of speech in surgical mask and KN95 mask conditions. We used the Visual Analogue Scale (VAS) with a straight line containing 100 points (1–100) with 1 and 100 representing ‘completely clear’ and ‘completely unclear’, respectively, that is, the higher the score, the less clear the speech sound.

#### Stimuli

The Rainbow Passage (Fairbanks, 1960) was used for listening tests (‘when the sunlight strikes raindrops in the air … the pot of gold at the end of the rainbow’). The stimuli represented non‐mask (*n* = 16), surgical mask (*n* = 12) and KN95 mask (*n* = 12) conditions. The sample size for the two mask conditions were *n* = 12 because four participants only recorded their voices with surgical mask and without KN95 mask. Therefore, using the same sample size for the two mask conditions would allow equal samples in statistical analyses. A total of 12 samples (30%) were repeated for intra‐rater reliability evaluation. In total, 52 samples were coded and randomized throughout for presentation to the listeners. All stimuli were normalized for intensity using the ‘normalize’ command in Audacity with the checkbox ‘normalize peak amplitude to –3.0 dB’ being checked. After normalization, the output intensity level of stimuli was between 72.0 and 75.0 dB SPL as measured in Praat, and was presented to listeners via a headphone. Normalization allowed stimuli to be presented at consistent intensity levels, avoiding potential impact of variable intensity levels on auditory–perceptual ratings.

#### Procedure

Listening tasks were conducted using an online auditory–perceptual rating tool called Bridge2practice, which is an online education and research platform developed for perceptual learning and practice of allied health professionals and students (Madill et al., 2019). Raters were required to listen to the speech stimuli as many times as they wished using headphones with volume adjusted to their most comfortable hearing level and make a judgment about speech sound clarity by changing the position of the slider on the VAS line described above. The instructions provided to the raters were as follows: ‘listen to the voice sample. It can be played as many times as you wish. Put the slider on a position in the line to indicate the degree to which you judged the level of clarity of the speech sound in the sample’. All stimuli were presented in randomized orders. Raters were not aware that some speech samples were produced with wearing either a surgical or KN95 mask. Responses were registered in the rating platform and exported to an Excel spreadsheet for analyses.

#### Reliability of perceptual ratings

Intra‐ and interrater reliability were assessed using SPSS 24.0 (SPSS, Inc., Chicago, IL, USA). Intraclass correlation coefficients (ICCs) were used to determine the level of agreement between the first and second (repeated) ratings (intra‐rater reliability) and across listeners (interrater reliability). ICC was calculated using a two‐way mixed model, consistency type and single measure analysis [ICC (3, 1)]. Intra‐rater reliability ranged from ICC = 0.647 to 0.785 for single measures and from ICC = 0.785 to 0.880 for average measures. Interrater reliability amongst the seven raters was moderate based on average measures (ICC = 0.692, *p* = 0.003).

### Statistical analyses

Data were managed in Microsoft Excel 365 and analysed using IBM SPSS Statistics v.24.0 (IBM Corp, 2018) and Prism v8.1.2 (GraphPad Software, 2018). Prior to analyses, normal distribution of the data was examined using Kolmogorov–Smirnov tests (Massey, 1951). Changes in the acoustic and perceptual measures across the three conditions (non‐mask, surgical mask and KN95 mask) were analysed using a linear mixed model with participants as random effects and condition and gender as fixed effects. Interaction between condition and gender was calculated. The mixed model was used as this study was not designed to test the effects of these masks given the design of the study in which we did not strictly control for between‐ and within‐subject variability in phonation, hence it was not possible to completely attribute the findings to masks’ effects. The mixed model that detected the linear trend of measures across the three conditions would be relevant. Parameter estimate utilized regression coefficient (*b*) for each effect associated with its 95% confidence interval (CI) and the *p*‐value. Significant fixed effects of conditions were further tested using pairwise comparison with Sidak adjustment for the *p*‐values. Multiple linear regression was used to examine the relationship between the acoustic measures and speech clarity ratings. Bonferroni adjustment was used where there were multiple correlation calculations. Significance level was *p* < 0.05 after any adjustment.

## RESULTS

### *A*_RMS_ of fricatives

**FIGURE 2:**
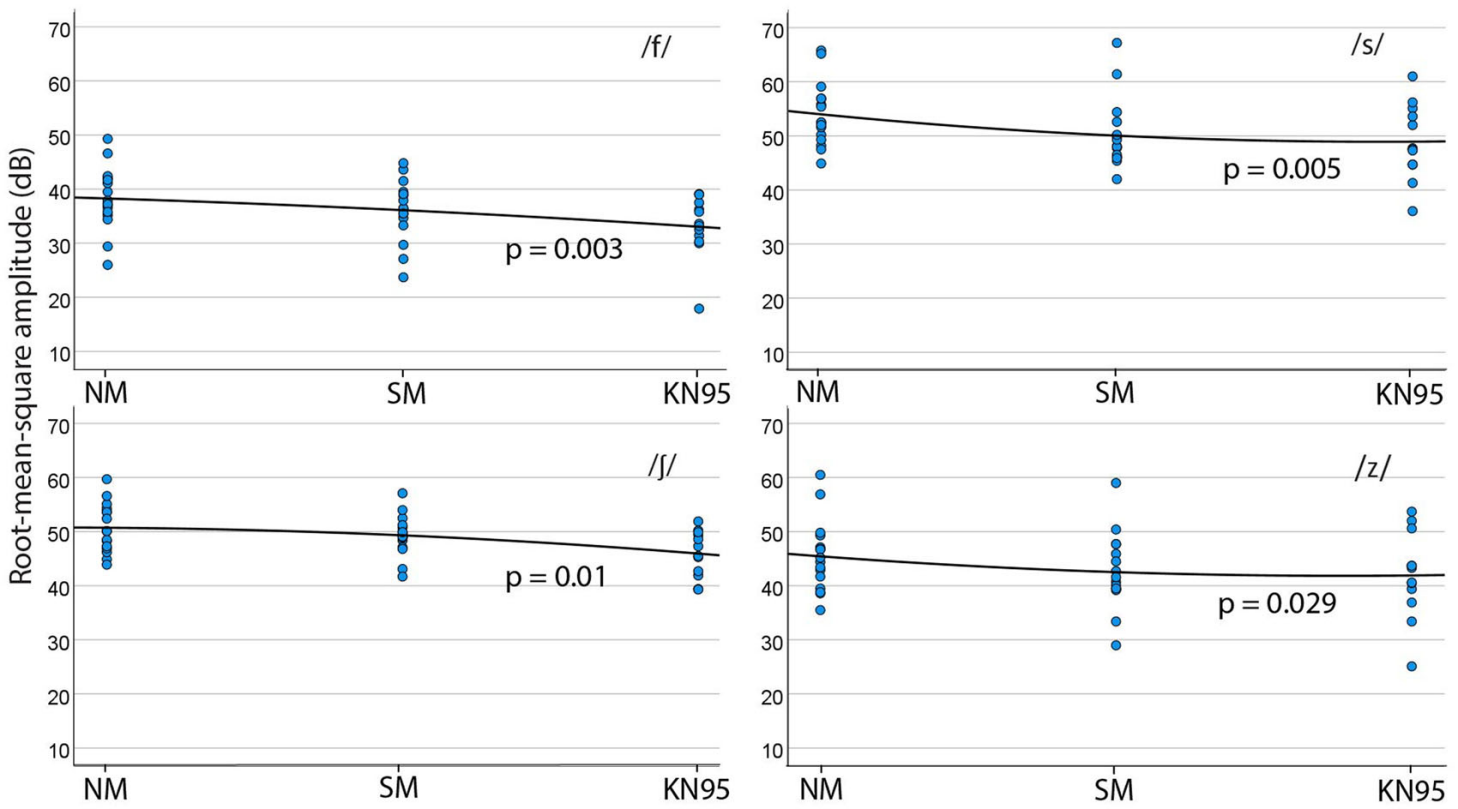
Root mean square (RMS) amplitude of all four fricatives in all conditions. The trend line is shown for each fricative. The *p*‐values represent the significant levels of parameter estimate of the decrease in RMS amplitude in the KN95 mask compared with non‐mask. NM, non‐mask; and SM, surgical mask [Colour figure can be viewed at wileyonlinelibrary.com]

This measure was obtained in Praat as Pascal units and converted to dB using the formula (2). Figure 2 shows *A*_RMS_ data and Table 2 shows results of linear mixed analyses for the fricatives. Table 2 shows significant effects of conditions for all the fricatives while there was no significant interaction between conditions and gender. This table also shows significant linear trend in *A*_RMS_ when phonation changed from non‐mask to KN95 mask.

**TABLE 2:** Results of linear mixed model analysis for the four fricatives

**Fricatives**	**Main effects of conditions**	**Condition × gender interaction**	**Parameter estimate (*A*_RMS_ in KN95 as compared with non‐mask)**
/f/	[*F*(2, 29.09) = 6.250, *p* = 0.006]	(*p* = 0.671)	*b* = 5.222, *t* = 3.241, *p* = 0.003
/s/	[*F*(2, 28.73) = 9.592, *p* = 0.001]	(*p* = 0.458)	*b* = 4.68, *t* = 3.008, *p* = 0.005
/ʃ/	[*F*(2, 29.19) = 8.424, *p* = 0.001]	(*p* = 0.529)	*b* = 5.128571, *t* = 4.249, *p* = 0.01
/z/	[*F*(2, 28.28) = 12.931, *p* < 0.001]	(*p* = 0.061)	*b* = 2.393, *t* = 2.301, *p* = 0.029

In /f/, phonation with KN95 mask resulted in a decrease of mean of 5.66 dB (95% CI = 1.58–9.74, Sidak‐adjusted *p* = 0.004) compared with non‐mask phonation. Meanwhile, speaking with a surgical mask showed a decrease in mean of 2.81 dB (95% CI = –0.88 to 6.5) compared with non‐mask, but this was not statistically significant (Sidak‐adjusted *p* = 0.179).

For /s/, as phonation changed from non‐mask to surgical mask and KN95 mask, mean of this measure (dB) decreased by 4.11 (95% CI = 0.55–7.67, Sidak‐adjusted *p* = 0.02) and 6.57 (95% CI = 2.63–10.51, Sidak‐adjusted *p* = 0.001), respectively. When phonation changed from surgical mask to KN95 mask, this measure decreased by mean of 2.46 dB (95% CI = –1.48 to 6.39) but this was not statistically significant (*p* = 0.329).

For /ʃ/, compared with non‐mask, mean of this measure decreased by 1.92 dB in surgical mask (95% CI = –0.84 to 4.69, non‐significant at Sidak‐adjusted *p* = 0.244) and by 4.95 dB in KN95 mask (95% CI = 1.89–8.01, Sidak‐adjusted *p* = 0.001). Compared with surgical mask, mean of this decreased by 3.03 dB (95% CI = –0.02 to 6.09) in KN95 and this was not significant (*p* = 0.052).

In /z/, when phonation changed from non‐mask to surgical mask and KN95 mask, mean *A*_RMS_ decreased by 3.43 dB (95% CI = 1.06–5.79, Sidak‐adjusted *p* = 0.003) and 4.98 dB (95% CI = 2.34–7.62, Sidak‐adjusted *p* < 0.001), respectively.

### Spectral moments of fricatives

**FIGURE 3:**
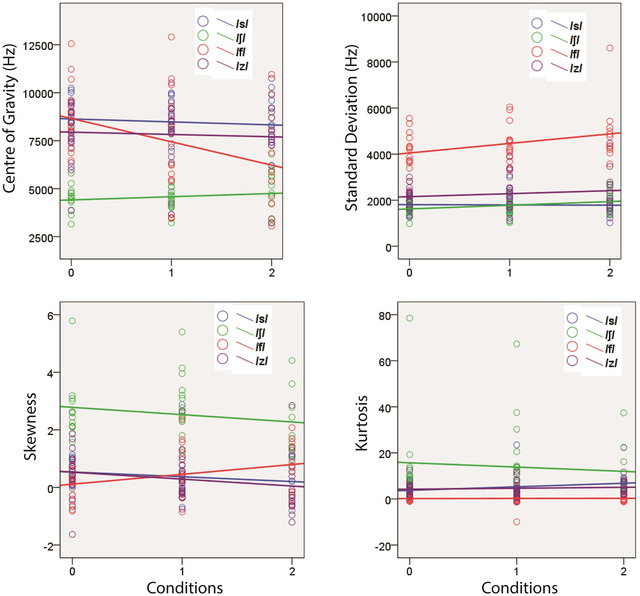
Spectral moments of the four fricatives in all conditions including a trend line for each fricative. 0 = Non‐mask, 1 = surgical mask and 2 = KN95 [Colour figure can be viewed at wileyonlinelibrary.com]

Figure 3 shows the four spectral moments of all four fricatives. It shows that the spectral moment measures of all fricatives except /f/ were consistent across conditions. For /f/, there was variation across conditions for its centre of gravity.

#### Centre of gravity

Table 3 shows results of fixed effects *F*‐test for all the fricatives. For centre of gravity of /f/, there was significant fixed effect of condition and the linear regression coefficient between non‐mask and KN95 were significant (*b* = 2859.36, *t* = 4.306, *p* < 0.001). Compared with non‐mask, the mean of this measure decreased by 1528.65 Hz in surgical masks (95% CI = 6.41–3050.89, Sidak‐adjusted *p* = 0.049) and by 1768.19 Hz in KN95 mask (95% CI = 86.57–3449.8, Sidak‐adjusted *p* = 0.037).

**TABLE 3:** Results of type III tests of fixed effects for spectral moments of four fricatives

**Fricatives**	**Centre of gravity**	**Standard deviation**	**Skewness**	**Kurtosis**
/f/	*F*(2, 28.82) = 4.684, *p* = 0.017*	*F*(2, 29.87) = 1.957, *p* = 0.159	*F*(2, 29.57) = 2.976, *p* = 0.066	*F*(2, 30.92) = 0.170, *p* = 0.845
/s/	*F*(2, 28.07) = 0.349, *p* = 0.709	*F*(2, 29.55) = 0.510, *p* = 0.606	*F*(2, 29.03) = 1.645, *p* = 0.211	*F*(2, 29.87) = 2.660, *p* = 0.087
/ʃ/	*F*(2, 28.36) = 1.004, *p* = 0.379	*F*(2, 27.36) = 2.869, *p* = 0.074	*F*(2, 28.71) = 2.928, *p* = 0.070	*F*(2, 28.35) = 3.326, *p* = 0.050*
/z/	*F*(2, 27.88) = 0.110, *p* = 0.896	*F*(2, 29.06) = 3.621, *p* = 0.039*	*F*(2, 29.16) = 2.227, *p* = 0.126	*F*(2, 29.03) = 0.247, *p* = 0.783

*Notes*: *Significance at 0.05. Significant results are shown in bold.

Other fricatives did not show statistically significant linear trend in centre of gravity across conditions (Table 3).

#### Standard deviation (SD)

Table 3 shows significant effects of conditions on SD of /z/. However, the linear regression coefficient was not statistically significant (*b* = 19.00, *t* = 0.087, *p* = 0.931). This measure of /z/ increased by a mean of 572.15 Hz (95% CI = 21.58–1122.73) in KN95 compared with non‐mask (Sidak‐adjusted *p* = 0.04).

#### Skewness and kurtosis

There was no significant fixed effect of conditions on Skewness for all fricatives. There was a marginally significant fixed effect for Kurtosis of /ʃ/ (Table 3). However, the regression coefficient (*b*) was not statistically significant (*b* = 4.235, *t* = 1.227, *p* = 0.230). There was a difference of mean of 8.902 (95% CI = 0.15–17.65, Sidak‐adjusted *p* = 0.05) between surgical mask and KN95 mask. No significant differences in this measure were observed between non‐mask and mask‐wearing conditions (*p* > 0.05).

### Amplitude of the first three formants

**FIGURE 4:**
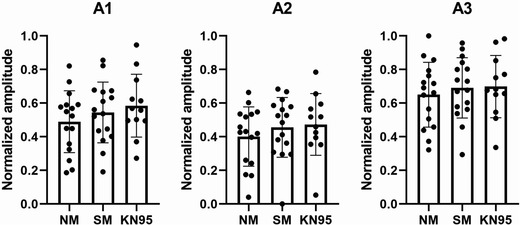
Normalized formant amplitude of the Rainbow Passage. Error bars indicate standard deviation. Dots represent individual values. NM, no mask; and SM, surgical mask

Figure 4 shows formant amplitudes A1, A2 and A3 measured from the Rainbow Passage reading. As there was great between‐speaker variability in these measures across conditions, all formant amplitude data were normalized so that they fell between 0 and 1. There were no significant fixed effects of conditions on A1 of Rainbow Passage (*p* = 0.273), A2 or Rainbow Passage (*p* = 0.540) and A3 of Rainbow Passage (*p* = 0.798).

**FIGURE 5:**
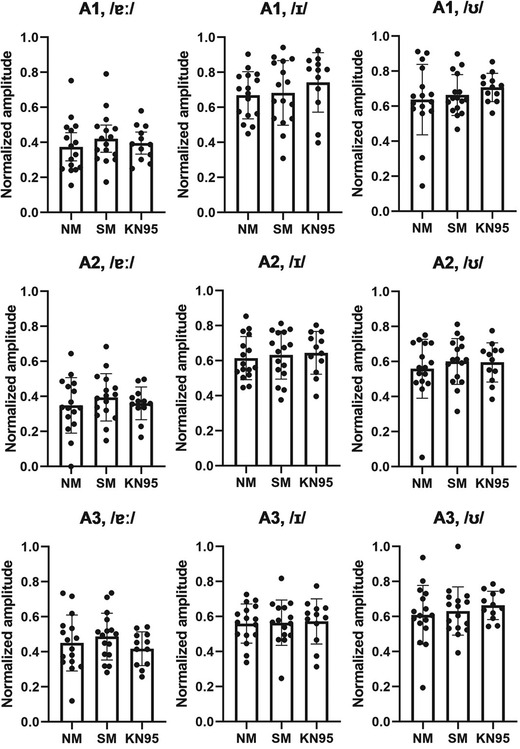
Normalized formant amplitude of three extracted vowels. Error bars indicate standard deviation. Dots represent individual values. NM, no mask; and SM, surgical mask

Figure 5 presents the normalized amplitudes A1, A2 and A3 for the three extracted vowels. For /ɐː/, no significant effects were observed for A1, A2 and A3 (*p* > 0.05). For /ɪ/, no significant effects were found for A1, A2 and A3 (*p* > 0.05). For /ʊ/, there were also no significant effects for A1, A2 and A3 (*p* > 0.05). Owing to non‐significant effects, no further post‐hoc analyses were implemented to clarify the interaction between condition and vowel amplitude.

Given the impact of vocal intensity on formant amplitude (Huber et al., 1999) and to evaluate within‐speaker variability in phonation, vocal intensity data were collected and mixed‐effects analysis showed no statistically significant differences across conditions (*p* = 0.109). Mean (SD) values of vocal intensity of the Rainbow Passage were 58.68 (4.91) dB, 60.7 (5.02) dB and 60.93 (5.06) dB for non‐mask, surgical and KN95 mask, respectively.

### Speech clarity ratings

**FIGURE 6:**
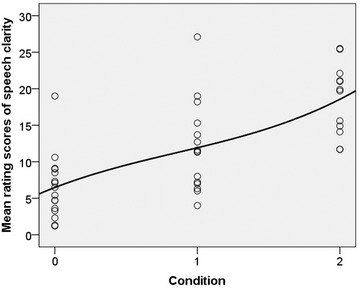
Rating score of speech clarity in three conditions including a trend line (rating scale 1–100, higher scores represent poorer speech clarity). 0 = Non‐mask, 1 = surgical mask and 2 = KN95

Rating scores were averaged from all raters and used to calculate the population's parameters and analysed statistically. Figure 6 shows rating scores of speech clarity of the Rainbow Passage across the three conditions with higher scores representing less clear speech. Mixed model analysis showed significant effects of conditions [*F*(2, 43) = 16.317, *p* < 0.001] and no significant interaction between conditions and gender (*p* = 0.883). The linear regression coefficient (*b*) of rating scores of speech clarity were statistically significant in both surgical mask (*b* = –6.62, *t* = –2.951, *p* = 0.005) and KN95 mask (*b* = –11.61, *t* = –5.279, *p* < 0.001). Compared with the non‐mask condition, mean speech clarity score in surgical mask increased by 5.87 (95% CI = 0.79–10.96, Sidak‐adjusted *p* = 0.019) and mean speech clarity score in KN95 mask increased by 12.55 (95% CI = 7.09–18.02, Sidak‐adjusted *p* < 0.001). Compared with surgical mask condition, mean speech clarity score in KN95 increased by 6.68 (95% CI = 1.19–12.17, *p* = 0.013).

### Relationship between acoustic measures and speech clarity ratings

Significant correlation was observed between clarity ratings and *A*_RMS_ of /f/ (*r* = –0.39, *p* = 0.01, significant after Bonferroni adjustment), /s/ (*r* = –0.481, *p* = 0.001, significant after Bonferroni adjustment), /ʃ/ (*r* = –0.540, *p* = 0.0002, significant after Bonferroni adjustment), and /z/ (*r* = –0.375, *p* = 0.013, non‐significant after Bonferroni adjustment). There was no significant correlation between clarity ratings and all formant amplitudes A1, A2 and A3 from the Rainbow Passage and all three vowels /ɐː/, /ɪ/, and /ʊ/ (*p* > 0.05).

Linear regression was calculated to examine the relationship between the acoustic measures and speech clarity ratings. Amplitude of fricatives were input into multiple linear regression equations based on the place of articulation. When *A*_RMS_ of /f/ was used as independent variable, it significantly predicted clarity rating (*b* = –0.456, *t* = 2.715, *p* = 0.0096). When *A*_RMS_ of /s/, /ʃ/, and /z/ were independent variables, only /ʃ/ was significant predictor (*b* = –0.74, *t* = 2.54, *p* = 0.015).

When formant amplitude measures of the Rainbow Passage were input as predictors, only A1 was the significant predictor (*b* = 1.957, *t* = 2.986, *p* = 0.0049) while A2 (*p* = 0.13) and A3 (*p* = 0.12) were not significant predictors of speech clarity ratings.

## DISCUSSION

The present study demonstrated that the RMS amplitude of fricatives obtained from speech samples produced with wearing either surgical mask or the KN95 respirator was lower than that in speech produced without a mask. These results supported Atcherson et al. (2017) who found that the total RMS of speech decreased in surgical masks, and Corey et al. (2020) who showed that KN95 respirators attenuated the acoustic energy of speech. The finding also agreed with Llamas et al. (2009) who found that speech produced with a surgical mask had lower fricative energy > 3.2 kHz than non‐mask speech. However, our findings disagreed with those reported by Asadi et al. (2020) who found that the RMS amplitude of speech was equal to or greater than that in non‐mask condition. Conflicting findings may result from differences in mask types used and experimental designs across studies. The decrease in voiceless fricative amplitude would add further levels of difficulty in understanding connected speech for people with hearing loss who have difficulties perceiving voiceless fricatives (Zeng & Turner, 1990). Discrimination of consonants in noise hearing environment would also be likely to be affected (Rahne et al., 2021).

The two mask types used in this study provide two different levels of barrier with KN95 mask having higher filtering levels than surgical mask (3M, 2020). This might contribute to the larger decrease in *A*_RMS_ of the fricatives in KN95 than in surgical mask. Asadi et al. (2020) have found that surgical mask or unvented KN95 respirators decreased the outward particle emission by 90% during speaking compared with non‐mask. Corey et al. (2020) showed that the attenuation properties of face masks were determined by factors such as material type, layer number, thickness, and weave patterns. They maintained that masks produced using more breathable materials would allow more acoustic transmission. They found that the three‐layer, 0.4 mm‐thick surgical mask (material: polypropylene) attenuated the acoustic signals by 3.6 dB while KN95 respirator (two layers, 0.6 mm thick) attenuated by 4.0 dB. These masks were equivalent to the masks we used in the present study. The above‐mentioned characteristics may stipulate how speech sounds are transmitted, absorbed, and deflected given that different materials have varying sound absorption effects due to varying transmission loss (a property of a material that relates to its sound attenuation characteristics) (Llamas et al., 2009). It is also likely that the acoustic energy might be deflected to the sides of the masks (Corey et al., 2020) where the microphone sensitivity is not the highest given the cardioid directionality and the placement position (45^o^ off the mouth axis) of the AKG C520 used in this study. The deflection effects, however, might be small as the mouth‐to‐microphone was only 6 cm. The lack of statistical significance of the difference in *A*_RMS_ of the fricatives between two masks (e.g., in /s/) suggested that the transmission loss of fricative noise could occur irrespective of mask specifications. The design of this study did not allow us to confirm the relationship between the material characteristics of the masks and the acoustic outcomes.

We hypothesized that the amplitudes of the second and/or third formants were different between the conditions as they are within the high frequency range. However, the findings rejected this hypothesis. All formant amplitude measures (A1, A2 and A3) of connected speech (Rainbow Passage) and all the edited vowels were not significantly different between non‐mask and mask conditions. These findings agreed with a previous report that vowel energy in the 1–8 kHz region did not decrease while wearing face masks (Nguyen et al., 2021). The findings also helped clarify which spectral components contribute to the decreased energy levels in the high frequency regions of speech produced with a face mask as mentioned in previous studies (Goldin et al., 2020; Magee et al., 2020). In particular, in the high frequency ranges, vowel amplitudes might not contribute to the decreased spectral levels of connected speech in mask wearing. However, these findings did not agree with Llamas et al. (2009) who found that surgical mask resulted in lower amplitude of vowel formants > 3.2 kHz. More research is needed to clarity the effects of medical masks on the vowel signals.

The discrepancy in the findings between fricative amplitudes and vowel amplitude suggested the selective presentation of different components of the speech spectra while wearing a mask. In other words, not all types of the speech signals are transmitted/absorbed/reflected similarly through face masks. This might be partially related to the amplitude levels of different phonemic types. In connected speech, vowels often have stronger power levels than voiceless fricatives for example, /θ/ and /f/ (Behrens & Blumstein, 1988a). That means they would be more successfully transmitted via the mask than the fricatives. The interaction between the mask materials and the phonetic content might also be a factor and would need more experiments to investigate.

This study found that speech clarity rating scores were steadily higher when phonation changed from non‐mask to both surgical and KN95 masks. Given that the raters were not informed that the speech samples included both non‐mask and mask conditions, the findings indicated that speech was less clear during mask conditions. It is possible that the decreased amplitudes of the included fricatives contributed to this perceptual result. We found significant relationship between clarity ratings and *A*_RMS_ of the fricatives /s/ and /ʃ/, and the significant prediction of *A*_RMS_ of some fricatives (/f/ and /ʃ/) and A1 of the Rainbow Passage, suggesting that these were amongst the factors contributing to speech quality in mask‐wearing. In addition, we also found that centre of gravity (Hz) of /f/ was lower in mask conditions. This reflected possible interaction between the masks and fricative signals and/or labial movement (/f/ is a labiodental fricative) leading to changes in quality of fricative production and causing misperception of segmental units in masked speech. Llamas et al. (2009) found misperceptions of speech produced with a face mask. They found that listeners confused between stop consonants (e.g., /t/∼/θ/) and misperceived the place of articulation of stop consonants and place of articulation of fricatives for example, /f/∼/θ/. The speech material and design of the present study did not allow confirmation of misperception of the segmental units, and this should be examined in the future.

Although the findings reasonably indicated that speech sound was less clear when the mask was on, it is not possible to confirm that these masks were completely responsible for the reduced speech clarity given the design of the study did not strictly control for potential confounding factors. Within‐speaker variabilities in phonation are unavoidable in speech production. However, some acoustic findings would suggest participants indeed did not use the clear speech style nor significantly changed their vocal effort. The change in centre of gravity in /f/ did not reflect the trend toward hyper‐articulation in clear speech style. It has been found that centre of gravity values of fricatives, including /f/, increase significantly in the clear speech speaking style (Maniwa et al., 2009). The absence of systematic/consistent statistical linear trends in the spectral moments across conditions did not support the occurrence of ‘clear speech’ given that the speaking style would result in modification of fricative characteristics (Maniwa et al., 2009). The lack of differences in A1, A2 and A3 between non‐mask and mask conditions also implied that the participants did not change vocal effort significantly while wearing a mask as it has been shown that these formant amplitudes increased with increased vocal effort (Lienard & Di Benedetto, 1999). Lastly, vocal intensity levels across conditions were not significantly different, implying the less likelihood of changing phonation behaviours significantly in mask conditions. As such, variability in phonation or speaking style would not be the significant factor that impacted on the auditory–perceptual result.

It is necessary to acknowledge that the term ‘speech clarity’ can have different meanings and implications and does not only imply a link with consonant production. However, the findings would suggest that improving fricative production might be a reasonable strategy for face mask users to compensate for the lost fricative information. In examining three different speaking styles that is, casual, clear, and emotional speech with and without a fabric mask, Cohn et al. (2021) found that speaking style outperformed the impact of face masks on intelligibility. They showed that a clear speaking style improves intelligibility of speech in mask condition.

This study had limitations which means it should be cautious in interpreting the findings. First, we only used some fricative consonants that may not be the only segmental units to play important role in speech recognition and to show changes in acoustic characteristics in mask wearing. Findings could not be generalized to other fricatives, consonants, and vowels which should be examined in the future.

This study did not control for within‐ and between‐speaker variability in phonation across the three conditions. It was not possible to attribute the findings entirely to the impact of the masks given the design of this study (real world, clinical data collection without strict laboratory‐controlled settings). Investigating absorption characteristics of materials was beyond the scope of this study. Future studies should test the effects of specific mask types and/or material characteristics on the speech signals.

We acknowledged that the term ‘speech clarity’ is poorly defined. We used similar methods as previous studies investigating speech clarity and made similar assumptions as to the meaning of the term. Our perceptual data was collected using an online rating tool where raters might have used different volume settings, which might affect the results. Future studies should use volume controlling apps that allow listeners to follow the required intensity settings.

## CONCLUSIONS

The following points can be made based on the findings of this study:

- There were significant decreases in spectral amplitude of fricative consonants /f/, /s/, /ʃ/, and /z/ while wearing either a surgical or KN95 medical mask. Amongst the spectral moments, only centre of gravity of /f/ decreased significantly in both mask conditions. Other spectral moment measures of all fricatives did not show systematic, significant trend across the conditions. The decreased fricative amplitude has implications for speech perception in communication between clinical staff and between HCWs and patients in clinics.
- Amplitude of the first three formants measured from the Rainbow Passage and the edited vowels /ɐː/, /ɪ/, and /ʊ/ was not significantly different across conditions. This implied that different phonemic units are manifested differently in speech produced in mask‐wearing. This also might suggest the selective effects of mask materials on the speech signals that this study was not designed to test.
- Rating scores of speech clarity were significantly different between non‐mask and mask‐wearing, the speech sound was perceived as significantly less clear in mask conditions. In the assessment and treatment of speech and voice disorders, it is important to consider the quality of the speech sound when both the clinician and the patient are wearing a face mask.

## CONFLICTS OF INTEREST

There were no conflicts of interest in this study. The authors are responsible for the content of this paper.

## Data Availability

The data that were used in this study are available from the corresponding author upon request.
